# The complete mitochondrial genome of the flea *Ceratophyllus wui* (Siphonaptera: Ceratophyllidae)

**DOI:** 10.1080/23802359.2018.1456370

**Published:** 2018-03-28

**Authors:** Liangfei Tan, Xuhua Guan, Lingyao Zhang, Fen Zhu, Chaoliang Lei

**Affiliations:** a Hubei Insect Resources Utilization and Sustainable Pest Management Key Laboratory, College of Plant Science and Technology, Huazhong Agricultural University, Wuhan, China;; b Hubei Provincial Center for Disease Control and Prevention, Wuhan, China

**Keywords:** *Ceratophyllus wui*, flea, Ceratophyllidae, mitochondrial genome

## Abstract

The complete mitogenome sequence of the flea, *Ceratophyllus wui* (Siphonaptera: Ceratophyllidae), was sequenced. The 18,081 bp long genome has the standard metazoan complement of 37 genes. These genes contain 13 protein-coding genes, 22 transfer RNA genes, two ribosomal RNA genes, and one control region. The nucleotide composition of the *C. wui* mitogenome was A: 37.72%, T: 38.99%, G: 9.51% and C: 13.78%. The A + T content is 76.71%, showing strong AT skew. Phylogenetic analysis indicated that Siphonapteran has sister relationship with Dipteran.

## Introduction

Fleas are vectors that parasitize on mammals and birds by blood-sucking. Moreover, they can evolve with theria (Zhu et al. 2015). The identification of flea is arduous merely depending on morphological characteristics (Yssouf et al. 2014). Mitochondrial DNA (mtDNA) sequences are essential to species identification and a deeper understanding of evolution. The flea *Ceratophyllus wui* belongs to the family of Ceratophyllidae in the order of Siphonaptera which was first collected from *Collocalia brevirostris innominatus* reported in 1996 (Wang and Liu 1996). Here, we elucidate the mtDNA genome of *C. wui*.

In this study, specimens were collected from the Swallow Cave of Tianyan Scenic in Hongping Town of Shennongjia (Hubei Province, China) which were located closely to where *C. wui* were recorded (31°15′ to 31°57′N, 100°56′ to 110°58′E). The specimens now were stored in Hubei provincial center for disease control and prevention museum. Next-generation sequencing (NGS) technology was used to obtain the complete mitogenome of this flea.

The complete mitochondrial genome of *C. wui* is a closed circular molecule 18,081 bp in length (GenBank accession number MG886872) and complement of 37 genes. These genes contain 13 protein-coding genes, 22 transfer RNA genes, two ribosomal RNA genes, and one control region (Table 1). The nucleotide composition of the *C. wui* mitogenome was A: 37.72%, T: 38.99%, G: 9.51%, and C: 13.78%. The A + T content is 76.71% with obvious AT skew, which was similar with that of the flea *Dorcadia ioffi* (Xiang et al. 2017).

**Table 1.** Genes coded by *Ceratophyllus wui* mitochondrial genome.

Gene	Direction	Location	Size (bp)	Anticodon	Startcodon	Stopcodon
tRNA^Ile^	F	1–64	64	GAT		
tRNA^Gln^	R	69–137	69	TTG		
tRNA^Met^	F	137–203	67	CAT		
NAD2	F	204–1217	1014		ATT	TAA
tRNA^Trp^	F	1216–1282	67	TCA		
tRNA^Cys^	R	1289–1349	61	GCA		
tRNA^Tyr^	R	1350–1413	64	GTA		
COX1	F	1411–2946	1536		ATC	TAA
tRNA^Leu^	F	2951–3014	64	TAA		
COX2	F	3016–3696	681		ATG	TAA
tRNA^Lys^	F	3699–3768	70	CTT		
tRNA^Asp^	F	3768–3832	65	GTC		
ATP8	F	3842–4000	159		ATA	TAA
ATP6	F	3994–4668	675		ATG	TAA
COX3	F	4668–5450	789		ATG	TAA
tRNA^Gly^	F	5451–5513	63	TCC		
NAD3	F	5511–5864	354		ATA	TAA
tRNA^Ala^	F	5867–5930	64	TGC		
tRNA^Arg^	F	5930–5992	63	TCG		
tRNA^Asn^	F	5990–6055	66	GTT		
tRNA^Ser^	F	6056–6123	68	TCT		
tRNA^Glu^	F	6124–6187	64	TTC		
tRNA^Phe^	F	6186–6250	65	GAA		
NAD5	R	6198–7925	1728		ATT	TAA
tRNA^His^	F	7969–8031	63	GTG		
NAD4	R	7967–9301	1335		ATG	TAA
NAD4l	R	9361–9654	294		ATG	TAA
tRNA^Thr^	F	9657–9722	66	TGT		
tRNA^Pro^	R	9723–9785	63	TGG		
NAD6	F	9797–10303	507		ATT	TAA
COB	F	10304–11443	1140		ATG	TAA
tRNA^Ser^	F	11450–11513	64	TGA		
NAD1	R	11533–12462	930		ATA	TAA
tRNA^Leu^	R	12479–12541	63	TAG		
rrnL	R	12521–13759	1239			
tRNA^Val^	R	13839–13907	69	TAC		
rrnS	R	13907–14686	780			
Control region	F	14687–18081	3395			

**Figure 1.**
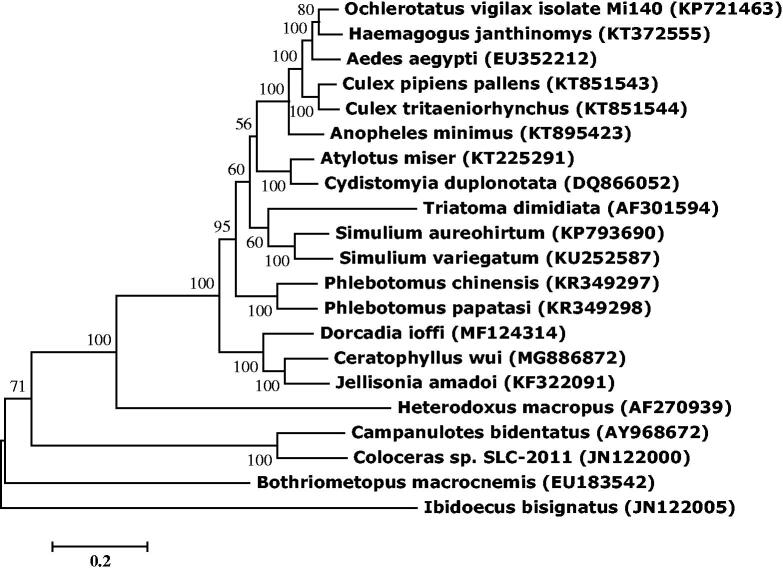
Molecular phylogeny of *C. wui* and other blood sucking insect species based on the complete mitochondrial genome. The complete mitochondrial genome was downloaded from GenBank and the phylogenic tree was constructed by maximum likelihood method with 1000 bootstrap replicates.

Molecular Evolutionary Genetics Analysis Version 6.0 (MEGA6.0) was used to construct phylogenetic tree with *C. wui* MtDNA and other blood-sucking insects with maximum likelihood method (Figure 1) (Tamura et al. 2013). *Ceratophyllus wui* MtDNA was closely clustered with other two previously reported flea species *Jellisonia amadoi* and *D. ioffi* (Cameron 2015; Xiang et al. 2017). Phylogenetic analysis indicated that Siphonapteran may have a closer affinity with a branch of Dipteran.
